# Outcome of Phacoemulsification in patients with and without Pseudoexfoliation syndrome in Kashmir

**DOI:** 10.1186/1471-2415-12-13

**Published:** 2012-06-06

**Authors:** Aalia R Sufi, Tejit Singh, Asmat Ara Mufti, Mudassar H Rather

**Affiliations:** 1 Department of Ophthalmology, Government Medical College, Jammu & Kashmir, Srinagar, India; 2 Directorate of Health Services, Kashmir, India; 3 Department of Surgery, Government Medical College, Jammu & Kashmir, Srinagar, India

## Abstract

**Background:**

The aim of the study is to compare the outcome of phacoemulsification in patients with and without pseudoexfoliation syndrome in Kashmir.

**Methods:**

200 patients were prospectively evaluated and divided into 2 groups. Group 1 comprised 100 cases with pseudoexfoliation and Group 2 (control) 100 cases without pseudoexfoliation. Phacoemulsification with posterior chamber intraocular lens implantation was performed by 3 surgeons. Intraoperative and postoperative observations were made in both the groups at regular intervals upto 6 months. A chi square test was used for statistical analysis.

**Results:**

Patients with pseudoexfoliation were significantly older (P = 0.000), had harder cataract(P = 0.030) and smaller mean pupil diameter(P = 0.000) than the control group. Intraoperative complications were comparable between the 2 groups except the occurrence of zonular dehiscence which was seen in 7% patients of Group 1 compared to 0% in Group 2. Higher postoperative inflammatory response was seen in Group 1(P = 0.000). Decrease in intraocular pressure (IOP) at all postoperative measurements was more in Group 1(P = 0.000). The visual acuity was better in the control group in the early postoperative period (P = 0.029), however the final visual acuity at 6 months was comparable between the 2 groups.

**Conclusions:**

Phacoemulsification in presence of pseudoexfoliation necessitates appropriate surgical technique to avoid intraoperative complications. Pseudoexfoliation is associated with higher inflammatory response, significant postoperative IOP drop and satisfactory visual outcome.

## Background

The Pseudoexfoliation(PEX) syndrome is a systemic disorder of unknown etiology and is associated with an increased incidence of intraoperative complications [1]. In PEX syndrome, lysosomal proteinases destroy the normal basement membrane structure of the non-pigmented epithelium of the ciliary body and anterior lens capsule which loosens the zonule-lens capsule complex and causes adhesions between the zonules and non-pigmented epithelium [1,2]. The rotational and posterior forces created during nucleus emulsification may lead to total separation of these weakened zonules, resulting in vitreous loss. Other factors thought to contribute to the increased incidence of intraoperative complications during cataract surgery in eyes with PEX syndrome are poorly dilating pupils, corneal endothelial changes and blood-aqueous barrier breakdown [3-8]. We designed a prospective study to evaluate the results of phacoemulsification in patients of Kashmiri origin, with and without pseudoexfoliation syndrome.

## Methods

This case control study was conducted prospectively between 2006–2008 in the Postgraduate Department of Ophthalmology, Government Medical College, Srinagar, Kashmir, India. 200 patients were divided into two groups: Group 1 comprised 100 cases with pseudoexfoliation and Group 2 (control) 100 cases without pseudoexfoliation.

### Exclusion criteria

Glaucoma, subluxation of the lens, conditions predisposing to zonular weakness and increased inflammatory response postoperatively, uveitis, history of trauma, history of intraocular surgical procedure, corneal pathologies, complicated cataract, retinal pathology like age related macular degeneration(ARMD), diabetic retinopathy, retinal detachment; all the conditions that would influence the outcome of visual acuity following surgery were excluded from our study.

Complete clinical evaluation was done including age, sex, visual acuity with Snellen chart, intra-ocular pressure (IOP) by Goldmann applanation tonometry(AT) and fundus examination. Detailed slit lamp biomicroscopy under maximal mydriasis was performed to assess the type and grade of cataract, and presence of phacodonesis or zonulolysis. Diagnosis of PEX was based on the presence of fibrillin deposits on the pupillary margin, anterior lens capsule, or both. Intraocular lens (IOL) power calculation was done with SRK II formula.

Patients were admitted one day prior to surgery and were prescribed topical antibiotics one hourly. Pupil was dilated with cyclopentolate 1%, tropicamide 1%, phenylephrine 10% and dilatation was maintained with flurbiprofen 0.03%. All patients provided informed consent.

### Surgical technique

Phacoemulsification surgery was performed in all eyes under peribulbar/posterior subtenon anesthesia by 3 experienced surgeons.

After creation of a superior incision and filling of the anterior chamber with a viscoelastic substance, continuous curvilinear capsulorhexis was performed with a capsulotomy needle or a capsulotomy forcep. In case of poor pupillary dilatation, iris hooks were used or the pupil was stretched mechanically. Hydrodisection was performed to loosen capsule cortical attachments. Phacoemulsification of the lens nucleus was performed. The lens cortex was aspirated, the capsular bag was filled with a viscoelastic material and a one –piece polymethyl methacrylate posterior chamber foldable intra ocular lens (5.5-6 mm optic) was implanted in the uneventful cases. In cases with posterior capsule rupture (PCR) or zonular dehiscence (ZD), the IOL was implanted in the sulcus after anterior vitrectomy. In one case of zonular dialysis, a capsular tension ring (CTR) was used.

Intra operative complications documented were: posterior capsule rupture, phacodonesis, zonular dehiscence and vitreous loss(VL).

Postoperative observations made were: IOP measurement, early postoperative complications like striate keratopathy(SK), corneal edema, anterior chamber flare and cell response, fibrin in the anterior chamber, posterior synechiae, inflammatory membrane, any capsular change- i.e. capsular contraction or opacification and visual acuity.

Patients were prescribed topical antibiotic steroids (dexamethasone 0.1% or prednisolone 1% and ofloxacin 0.3%) one hourly for the first week and then tapered gradually over a period of four to six weeks. Mydriatic in the form of tropicamide 1% was prescribed if the need arose.

Post operative follow up examinations were conducted on day one, two weeks, four weeks, six weeks and six months under a definite proforma by the same observer.

Best corrected visual acuity(BCVA) was assessed at six months. Fundus examination was done and reason for poor visual acuity,if any, was looked into.

### Data processing and analysis

Statistical analysis of the data was done by using chi-square test (*χ*2 test) without or with Yates correction as and when required. The level of significance was fixed at P-value less than 0.05 i.e. P < 0.05 was taken to be statistically significant.

## Results

In our study, the mean age of patients with PEX was significantly higher (P = 0.000) than those in the control group that is 76% of cases with PEX were older than 60 years of age compared to 27% of cases of control . There was a statistical difference in the sex distribution, with male predominance, 68% of patients with PEX were males as compared to 51% in the control group (P = 0.014). Slit lamp assessment showed that combined form of cataract (cortical changes with nuclear changes with varying degree of posterior sub capsular cataract) was the predominant form of cataract operated in our study in either group. However patients with PEX had harder cataract with nuclear sclerosis ≥ grade 3(P = 0.030). 73% patients of Group 1 showed PEX material on the pupillary margin only and 27% showed PEX both on the pupillary margin and a central disc on the anterior lens capsule. Our study showed a highly significant difference in the preoperative pupillary dilatation in response to mydriatics between the two groups (Table 1).

**Table 1:** Distribution of patients on the basis of Pre-operative Pupil Dilatation

**Pupil**	**PEX**	**Control**	**Total**
**Dilatation**	**Frequency**	**Frequency**	**Frequency**
Max(> = 5 mm)	10	95	105
Mid(3-5 mm)	85	5	90
Min(<3 mm)	5	0	5
Total	100	100	200
χ2=144.921, P=0.000, p<0.05,		**Highly Significant**	

**Observations:** The difference in the preoperative pupil diameter after maximal mydriasis was highly significant between the two groups, with 85% eyes with PXE attaining pupil size between 3 to 5 mm whereas 95% eyes of the control group with pupil > =5 mm.

In our study, the rates of PCR and VL were comparable in both the groups. PCR occurred in 7%, VL in 2% cases of Group 1 and 8% and 1% in Group 2 respectively, however, the rate of ZD was greater (7%) in the PEX group whereas no patient in the control group developed ZD. In 6% patients with PEX, lens was implanted in the sulcus compared to 2% in the control group (Table 2). Capsular tension ring (CTR) was implanted in one patient with PEX who developed ZD intraoperatively, in order to stabilize the bag.

**Table 2:** Distribution according to Intra operative complications

**Complications**	**PEX Frequency**	**Control Frequency**	**Total Frequency**
PCR	7	8	15
SF lens	6	2	8
ZD	7	0	7
VL	2	1	3
No lens implanted	1	1	2
Total	23	12	35
χ2=5.573 P=0.134, p>0.05,		**Not significant**	

**Observations:** As per intraoperative complications, there was no statistical difference between the two groups However, comparison of ZD and SF lens showed highly significant difference between the groups.

More eyes in Group 1 had a significantly (P = 0.00) higher postoperative inflammatory response in the form of anterior chamber flare, corneal edema and inflammatory membrane (Table 3).

**Table 3:** Distribution according to postoperative Slit lamp Examination (SLE)

**Findings**	**PEX Frequency**	**Control Frequency**	**Total Frequency**
Clear pseudophakia	32	72	104
SK	22	22	44
Flare	30	1	31
Corneal Edema	12	3	15
Corneal burn	0	1	1
Hyphaema	0	1	1
inflammatory membrane	4	0	4
Total	100	100	200
χ2=46.154 P=0.000, p<0.05,		**Highly Significant**	

**Observations:** Eyes with PEX had greater inflammatory reaction with 30% having flare, 12% having corneal edema compared to 1% and 3% of the control group respectively. Result was statistically significant.

Early development of posterior capsule haze was seen in 2% of eyes with PEX as compared to 0% in the control group. At 4 weeks, the occurrence of posterior capsular opacity (PCO) had increased with 4% PCO in group I compared to 1% in group 2. However at 6 months, slit lamp examination revealed that the frequency of PCO was 8% in the cases with PEX and 4% in patients of the control group. The difference was not significant statistically. In our study, we noted that at presentation, 45% patients of Group 1 had IOP 18–20 mmHg whereas 69% patients of Group 2 had IOP in the range of 16–18 mmHg. However there was a significant decrease in IOP documented in the PEX group at 6 months postoperatively; 92% cases of the PEX group recorded an IOP <16 mmHg compared to 68% of the control group (Table 4).

**Table 4:** Distribution of patients according to IOP at 6 months

**IOP (mmHg)**	**PEX Frequency**	**Control Frequency**	**Total Frequency**
**8-10**	8	0	8
**10-12**	9	1	10
**12-14**	34	21	55
**14-16**	41	46	87
**16-18**	5	29	34
**18-20**	2	3	4
**20-22**	0	0	0
**22=>**	1	0	0
**Total**	100	100	100
χ2=35.90,p=0.000,p<0.05		**Significant**	

**Observations:** The difference in intraocular pressure was found highly significant between the two groups. 92% of the cases with PEX had IOP < 16 mm Hg as compared to 68% cases of the control group.

In the early postoperative period at two weeks, there was a statistically significant difference in the uncorrected visual acuity (UCVA) between the two groups (P = 0.029). However the difference in the UCVA at 6 months was not found to be statistically significant. The BCVA at 6 months postoperatively was 6/18 or better in 97% cases of the control group and 94% of cases with PEX (Table 5).

**Table 5:** Distribution according to BCVA at 6 months

**Visual Acuity**°	**PEX**	**Control**	**Total**
	**Frequency**	**Frequency**	**Frequency**
≤6/60	2	1	3
6/36-6/24	4	2	10
6/18-6/12	22	14	47
≥6/9-6/6	72	83	140
Total	100	100	200
χ2=3.558 P=0.313, p>0.05		**Not significant**	

**Observations:** The BCVA between the two groups was comparable, with 72% of PEX cases having BCVA ≥ 6/9 as compared to 83% of cases in control group.

## Discussion

Pseudoexfoliation Syndrome was first described by Lindberg in 1917 [9]. Several studies have reported differences between eyes with and without PEX. Our study was aimed at documenting this difference in patients from Kashmir.

Significantly more eyes in Group 1 had hard cataract i.e. Grade 3 to Grade 4. Also the nuclei were found to be harder intraoperatively at the time of phacoemulsification than the preoperative LOCS III classification. This was consistent with the study of Shastri and Vasavada [10].

We noted higher preoperative IOP in patients with PEX. This was consistent with previous studies – Shingleton et al. [11] and Damji [12] who reported a mean baseline IOP higher in PEX (17.60 mmHg) versus 16.08 mmHg in the control group.

A good mydriatic pupil is one of the main requirements for a safe and successful phacoemulsification surgery. This is even more important in eyes with PEX syndrome, in which surgery is more complicated because of the risks associated with loss of zonular integrity and poor pupillary dilatation [13]. In our study, the mean pupil diameter achieved after maximal mydriasis was significantly less in the PEX Group. This necessitated the use of iris hooks, mechanical pupil stretching and viscomydriasis to facilitate surgical maneuvers.

Cataract surgery in eyes with PEX syndrome is considered a challenge because of weakened zonules and poor pupillary dilatation. Initial case reports document increased rate of complications during cataract extraction in patients with PEX syndrome. Drolsum and co-authors [14] found a frequency of 9.6% of capsular tear, zonular tear or vitreous loss in eyes with PEX. In the study by Shingleton and co-authors [11], the rate of vitreous loss was 4% in the PEX eyes and 0% in non-PEX group.

However recent reports by Shastri and Vasavada [10], Michael Hyams et al. [15] and others report no significant difference in the rate of complications between patients with and without PEX. Raymond J. Nagashima [16] reported no cases of posterior capsular tears and zonulo-dialysis. In our study, though intraoperative complications were comparable between the two groups, the occurrence of zonular dehiscence and sulcus fixated lens was more in Group 1.

Various factors that explain the difference between our intraoperative result and the lesser complications in the recent reports are:

1. Surgical experience being a crucial factor. Comparatively we are in the initial phase of phacoemulsification. It is to be stressed here that the rate of complications was more in patients operated in the earlier part of our study whereas it was significantly less in the later part of the study. We attributed this to the increasing experience of surgeons which acted as a crucial factor when operating on eyes with PEX.

2. With experience we learnt that gentle multi-quadrant hydrodissection with very gentle in-the-bag nuclear rotation is advocated to avoid zonular stress and dehiscence in PEX cases.

3. It was also found that rather than doing in-the- bag nucleotomy with its inherent danger of further damaging the already weak zonules, it is safer to do a supra-capsular pulsed phaco with soft shell visco-technique to protect the corneal endothelium after dividing the nucleus into two halves as the stress on the zonules by this technique is much less, and all the maneuvers therein are outside the bag reducing any stress on the zonules.

4. Michael Hyams et al. [15] implanted an anterior chamber IOL in higher number of patients with PEX. This may act as a surrogate for complications.

5. Also degree of PEX was not graded in any of the studies. It was observed in grading the degree of PEX, intraoperative complications were seen more in cases with severe exfoliation i.e. PEX present on the lens capsule and zonules.

We noted a higher inflammatory response postoperatively in patients with PEX in the form of flare, cells, corneal edema and inflammatory membranes. The significantly higher postoperative inflammatory response in patients with PEX can be attributed to the transient breakdown of the blood-aqueous barrier that occurs during phacoemulsification in patients with PEX [17]. In addition iris vessels are pathological with an increased permeability for protein in eyes with PEX [18].

The literature has shown a decrease in IOP after phacoemulsification that is more pronounced in eyes with a higher preoperative IOP. It is speculated that phacoemulsification removes a source of PEX material (the anterior lens capsule) and results in or stimulates clearance of PEX and pigment debris from the anterior segment, in particular the trabecular meshwork [12]. This was supported by other studies by DJ Cimetta [19] and Shingleton [11]. Our study too showed a decrease in IOP after phacoemulsification which was more pronounced in patients with PEX than in controls thus being consistent with other studies.

The UCVA in the early postoperative period was significantly better in the control group as compared to the group with PEX. This was attributed to the higher postoperative inflammatory response which affected the visual acuity in patients with PEX. However the UCVA later was comparable between the two groups. This was attributed to the clearance of inflammatory reaction that had caused a significant drop in visual acuity previously. Though the difference in BCVA at 6 months between the two groups was not statistically significant yet visual acuity in the PEX group was less on account of the occurrence of posterior capsular haze and opacity which was more in this group. This was consistent with Akinci et al. [20] and Shastri and Vasavada [10] who reported no significant difference in the visual acuity gain between the two groups (p > 0.05). However Streho M [21] reported a mean postoperative visual acuity of 0.4 ± 0.6 LogMar in the PEX group and 0.2 ± 0.1 LogMar in the control group. Drolsum et al. [14] reported a visual acuity of 0.5 (6/12) or better achieved in 86.5% of eyes with PEX and 92.4% in control group (P = 0.02) at 4 months.

## Conclusions

PEX with cataract is a challenging situation with probable higher intraoperative complications as compared to other cataracts due to weak zonules, poor pupillary dilatation and harder nuclei.

Certain preoperative and intraoperative measures are imperative like the availability of iris hooks, CTR, good gentle complete hydrodissection, gentle in-the- bag nuclear rotation and preferably a supracapsular nucleatomy using a soft shell visco-technique and pulsed phaco power to avoid any inbag stress.

Keeping the above points in view, we found that phacoemulsification is safe and effective in eyes with PEX. It is associated with a significant postoperative drop in IOP. However, there is, in our view, significant postoperative inflammatory reaction in patients with PEX that affects the visual status in the early postoperative period. Also there is increased posterior capsular opacification in some of these cases. However, the final visual outcome was comparable between the two groups.

## Competing interests

The authors declare that they have no competing interests.

## Authors' contributions

ARS was involved in conception and design of draft, in acquisition of data, analysis and interpretation of data and in drafting the manuscript. TS was involved in revising the draft critically for important intellectual content; and gave final approval of the version to be published. AAM contributed in drafting the manuscript. MHR contributed in drafting the manuscript. All authors read and approved the final manuscript.

## Pre-publication history

The pre-publication history for this paper can be accessed here:

http://www.biomedcentral.com/1471-2415/12/13/prepub
